# Evolved and Plastic Gene Expression in Adaptation of a Specialist Fly to a Novel Niche

**DOI:** 10.1111/mec.17653

**Published:** 2025-01-09

**Authors:** Rachel A. Steward, Jesús Ortega Giménez, Shruti Choudhary, Oliver Moss, Yi Su, Olivier Van Aken, Anna Runemark

**Affiliations:** ^1^ Department of Biology Lund University Lund Sweden; ^2^ Cavanilles Institute of Biodiversity and Evolutionary Biology Universidad de Valencia Paterna Spain; ^3^ Department of Forest Genetics and Plant Physiology, Umeå Plant Science Centre Swedish University of Agricultural Sciences Umeå Umeå Sweden; ^4^ Department of Plant Breeding Swedish University of Agricultural Sciences Alnarp Lomma Sweden

**Keywords:** adaptation, coexpression, host shift, inversion, plasticity, Tephritidae, transcriptomics

## Abstract

How gene expression evolves to enable divergent ecological adaptation and how changes in gene expression relate to genomic architecture are pressing questions for understanding the mechanisms enabling adaptation and ecological speciation. Furthermore, how plasticity in gene expression can both contribute to and be affected by the process of ecological adaptation is crucial to understanding gene expression evolution, colonisation of novel niches and response to rapid environmental change. Here, we investigate the role of constitutive and plastic gene expression differences between host races, or host‐specific ecotypes, of the peacock fly *Tephritis conura* , a thistle bud specialist. By cross‐fostering larvae to new buds of their natal host plant or the alternative, novel host plant, we uncover extensive constitutive differences in gene expression between the host races, especially genes associated with processing of host plant chemicals. However, evidence for expression plasticity was minimal and limited to the ancestral host race. Genes with host race‐specific expression are found more often than expected within a large inversion in the *T. conura* genome, adding to evidence that inversions are important for enabling diversification in the face of gene flow and underscores that altered gene expression may be key to understanding the evolutionary consequences of inversions.

## 1 Introduction

Colonisation of novel environments is a fundamental driver of biodiversity, often resulting in locally adapted, ecologically divergent populations, ecotypes or species (Rundle and Nosil 2005). Uncovering the genomic changes underlying this process of adaptation is critical to understanding how the diverse selection pressures of a novel environment can lead to ecological divergence and, ultimately, speciation (Wolfsberger, Battistuzzi, and Oleksyk 2022). Five decades of comparative transcriptomics have clarified that sequence divergence at gene loci, especially within protein‐coding regions, is insufficient to explain major morphological, physiological and behavioural divergence between species. Rather, mechanisms regulating expression of these genes are likely to account for biological and ecological divergence among populations, ecotypes and species (Barrier, Robichaux, and Purugganan 2001; Bawin et al. 2024; Carroll 2000; King and Wilson 1975; Rivas et al. 2018; Wittkopp and Kalay 2012). Because small changes in genetic or epigenetic regulatory mechanisms can have large, cascading effects in coexpressed gene networks, significant expression differences can evolve between lineages adapting to different niches even over short evolutionary timescales, with large consequences for phenotypes under selection in new environments. Constitutive differences in gene expression, here defined as expression differences between ecotypes or incipient species that do not change with environment, may not only create important barriers to recolonisation of alternative niches but could also lead to regulatory changes that limit opportunities for gene flow between divergent lineages. In particular, hybrids between locally adapted or speciating populations may be unable to achieve the gene expression necessary to succeed in either niche. Thus, understanding the evolution of expression during colonisation and adaptation to novel niches is fundamental to our understanding of ecological speciation.

Both constitutive and plastic expression differences play a role in initiating and maintaining ecological divergence and local adaptation in novel environments (Albecker et al. 2021; Ballinger et al. 2023; Campbell‐Staton et al. 2017; Ghalambor et al. 2015; Mack et al. 2018; Schoville et al. 2012; Xu et al. 2023). Ancestral plasticity is an important source of phenotypic variation that can enable colonisation and ultimately divergent evolutionary adaptation to a novel niche (Gibert 2017; Janz and Nylin 2008; West‐Eberhard 2003). Strong selection on gene expression in the novel niche is predicted to produce genetic accommodation, a change in degree of ancestral plasticity (Crispo 2007; Pfennig and Ehrenreich 2014; West‐Eberhard 2003), or assimilation, a loss of ancestral plasticity in the derived lineage (Gibert 2017). Assimilation has been observed in both naturally and experimentally evolved populations that are adapted to divergent environments (Brennan et al. 2022; Kelly 2019; Wood et al. 2023). In contrast, Celorio‐Mancera et al. (2023) found that even specialist species like butterfly species retained gene expression patterns similar to generalist species and associated with feeding on a wide range of host plant families. Furthermore, in a reanalysis of published data, Chen and Zhang (2023) found that genetic assimilation is a rare outcome for ancestrally plastic genes. Thus, one major question about the evolution of gene expression during divergence on a novel niche is whether populations in the derived niche exhibit less adaptive transcriptional plasticity than populations exploiting the ancestral niche, as might be expected if strong selection in the derived niche has resulted in genetic assimilation.

Host plant shifts provide excellent systems for examining the role of gene expression in adaptation to novel environments. Colonisation of novel host plants and subsequent reproductive isolation, or even speciation, is especially common among herbivorous insects (Drès and Mallet 2002; Hernández‐Hernández et al. 2021; Jaenike 1990). Hernández‐Hernández et al. (2021) found that over 70% of reviewed sister species pairs were ecologically divergent, especially in their interactions with host plants. Many herbivorous insects are highly dependent on their host plants throughout their juvenile and adult lives (Ehrlich and Raven 1964; Janz 2011; Jermy 1984), and host plants represent complex, multidimensional niches with varied selection pressures affecting insect fitness. Host shifts are hypothesised to involve an initial period of niche polymorphism or plasticity before ecological specialisation and speciation (Drès and Mallet 2002; Janz and Nylin 2008; Nyman et al. 2010). In fact, major patterns of herbivorous insect diversification can be linked to host use variability, rather than abrupt switches between host plants (Braga et al. 2018), suggesting an important role for phenotypic plasticity in adaptation and speciation (i.e., the oscillation hypothesis, c.f. Janz and Nylin 2008). A secondary prediction of the oscillation hypothesis is that the gene regulatory networks facilitating diet plasticity, reacquisition of historical hosts or colonisation of additional hosts should be maintained, especially in populations continuing to use the ancestral host (Celorio‐Mancera et al. 2023; Janz and Nylin 2008). Counter to these predictions, however, many expression differences between closely related species feeding on different host plants are constitutive, fixed differences, not transcriptionally plastic (Birnbaum and Abbot 2020; Eyres et al. 2016; Orsucci et al. 2018; Silva‐Brandão et al. 2017). Few studies directly assess constitutive versus plastic transcriptional differences in ongoing ecological speciation (reviewed by Birnbaum and Abbot 2020), how expression differences relate to underlying genomic architecture and whether plasticity is likely to differ between ancestral and derived niches. Thus, the relative importance of constitutive and plastic expression differences in local adaptation and speciation on new host plants remains unclear.

**FIGURE 1:**
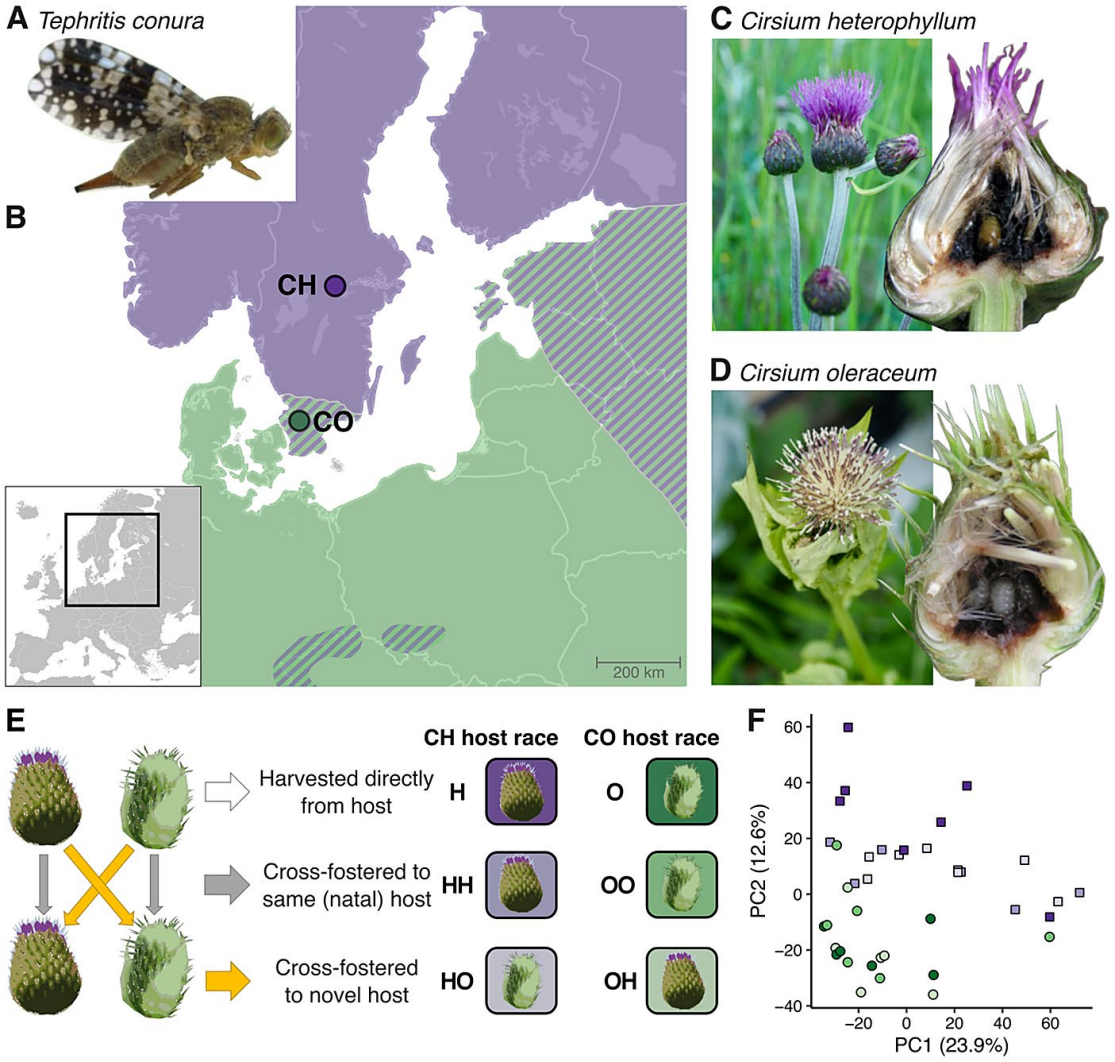
*Tephritis conura* host plants, sampling sites and cross‐fostering design. (A) Adult female *T. conura* . (B) Host plant distribution and sampling locations of thistle buds infested by flies specialising on *Cirsium heterophyllum* (CH) and *C. oleraceum* (CO); light purple = allopatric *C. heterophyllum* , light green = allopatric *C. oleraceum* and striped = sympatric regions. (C) *C. heterophyllum* buds, bisected bud showing a natural clutch of CH fly pupae. (D) *C. oleraceum* thistle buds, bisected bud showing a natural clutch of CO fly larvae. (E) Cross‐fostering design and resulting treatments. (F) Principal component analysis of the 5000 most variable genes expressed by third (final)‐instar larvae. The CH host race is shown with purple squares, and the CO host race with green circles and colour gradients are scaled according to the cross‐fostering design (Figure 1E). Images A, C, D: K.J. Nilsson; bisected bud images C, D: T. Diegisser; used with permission.

Here, we investigate the roles of gene expression divergence and plasticity underlying ongoing adaptation by some lineages of the peacock fly *Tephritis conura* to a novel host plant (Figure 1A). In continental Europe (Figure 1B), *T. conura* populations specialise on melancholy thistles ( *Cirsium heterophyllum* ; Figure 1C) or cabbage thistles (*C. oleraceum*; Figure 1D), laying eggs and spending larval and pupal stages within a single thistle bud (Romstöck‐Völkl 1997). Broad geographical analysis of mitochondrial haplotypes suggests that *C. heterophyllum* was the ancestral host, and *C. oleraceum* has been colonised evolutionarily recently (Diegisser, Seitz, and Johannesen 2006). Adult females prefer to oviposit in their specialised host, and flies developing in the wrong host experience highest mortality in the larval stage (Diegisser, Johannesen, and Seitz 2008; Nilsson 2024). Flies specialised in *C. heterophyllum* and *C. oleraceum* (CH and CO flies, respectively, in Figure 1E) have a linked genomic basis of host use and reproductive isolation in the form of a large putative inversion on the ancestral dipteran X chromosome (Steward et al. 2024). We thus refer to the *C. heterophyllum* and *C. oleraceum* specialist lineages as host races or ecotypes that parasitise different hosts and are in the process of speciation (c.f. Drès and Mallet 2002). However, whether expression differences important for adaptation to the divergent host thistles are associated with the putative inversion is unknown.

To understand the role of gene expression divergence in niche colonisation and incipient speciation, we test the extent to which expression differs between *T. conura* host races. Using a cross‐fostering design (Figure 1E), we further test whether *T. conura* flies can plastically adjust gene expression in response to feeding on the alternate thistle host and whether the ancestral or derived host races differ in the extent of their expression plasticity. Finally, we address how genomic architecture and signatures of selection are related to constitutive and plastic gene expression differences between *T. conura* host races. Differential gene expression analyses reveal extensive expression differences between the two host races feeding on their natal host species, but limited evidence for gene expression plasticity when switched to a novel host species. However, a gene coexpression network approach suggests that some coexpressed gene modules can be plastically expressed in the ancestral host race, but not in the derived host race. Finally, we find that constitutive expression differences between host races were especially likely to occur in genes within the highly divergent putative inversion, suggesting a concerted role for genetic architecture and gene expression evolution in *T. conura* speciation.

## 2 Methods

### 2.1 Sample Collection

In northern parts of Europe, populations of *Tephritis conura* (Figure 1A) oviposit in buds of the melancholy thistle, *Cirsium heterophyllum* (Figure 1C; typically 7–8 eggs), while in southern parts of the range, flies lay eggs in buds of the cabbage thistle, *C. oleraceum* (Figure 1D; ca. 6–7 eggs) (Janzon 1984; Romstöck‐Völkl 1997). The host plants belong to the same Eurasian thistle clade but are not sister species (Ackerfield et al. 2020; Bureš et al. 2023) and are expected to differ in defensive chemistry (Jordon‐Thaden and Louda 2003). Here, we refer to the divergent fly lineages as host races, defined as genetically differentiated populations that use different hosts but retain some degree of gene flow (Drès and Mallet 2002). We abbreviate the flies specialising in *C. heterophyllum* and *C. oleraceum* as CH and CO flies respectively. Although the ranges overlap broadly and there are few morphological differences (Nilsson et al. 2022), host races show considerable genomic divergence and reduced gene flow in contact zones both east and west of the Baltic Sea (Steward et al. 2024; Figure 1B). Fitness costs of using the wrong host are highest in the larval stage (Diegisser, Johannesen, and Seitz 2008), and we therefore focus on gene expression differences in third (final) instar larvae feeding on different host plants. Flies used in this experiment were sourced from a CH population in central Sweden (59.63  N, 14.58  E) where *C. heterophyllum* is allopatric, and a CO population in southern Sweden (55.90  N, 13.41  E) where the host plant ranges are broadly sympatric (Figure 1B). Direct sampling and cross‐fostering experiments were performed on *C. heterophyllum* and *C. oleraceum* plants in a common garden at Lund University, Sweden (Supplementary Methods 1 in Appendix S1).

To explore gene expression differences between the two host races, third‐instar larvae were extracted from infested buds of each host plant, frozen in liquid nitrogen and preserved at −80°C for RNA extraction. We used relative sizes to estimate larval instar. The flies sampled from the original host plants form two control groups: H and O (Figure 1E). For cross‐fostering treatments (Figure 1E), third‐instar larvae were extracted from infested buds. Half of the larvae were extracted and directly (within 1 min) switched into uninfested buds from the novel (i.e., nonadapted) host plant, one larva per bud, while the remaining larvae were directly switched to uninfested buds of another individual of the natal (i.e., adapted) host plant. This second group was included to control for gene expression differences resulting from the stress of moving larvae between thistle buds. After 6 h feeding in the new bud, larvae were extracted from the buds and preserved for RNA extraction. We confirmed that larvae fed on the new host by checking for the presence of feeding damage or frass. The result was four cross‐fostering treatments: CH larvae switched to *C. heterophyllum* buds (HH), CH larvae switched to *C. oleraceum* buds (HO), CO larvae switched to *C. oleraceum* buds (OO) and CO larvae switched to *C. heterophyllum* buds (OH) (Figure 1E; Table S1; Supplementary Methods 1 in Appendix S1).

### 2.2 RNA Extraction, Library Prep and Sequencing

RNA was extracted from individual flies using Sigma Aldrich's Plant RNA kit with on‐column DNAse treatment, which we found performs best with these flies. Illumina TruSeq stranded mRNA libraries were prepared by SciLifeLab (Stockholm, Sweden). Mature transcripts were enriched using poly‐A selection. Sequencing was performed by SciLifeLab using the NovaSeq6000 (NovaSeq Control Software 1.7.5/RTA v3.4.4; ‘NovaSeqXp’ workflow in ‘S4’ mode flowcell for 150 bp paired‐end reads). Within each treatment, we minimised the chance of sequencing siblings by selecting larvae originating from different buds, as *T. conura* emerging from the same flower head are mostly full siblings when flower resources are plentiful (Romstöck‐Völkl and Wissel 1989). We aimed to sequence one individual from each family in each treatment for a total of seven families within each host race.

Raw read quality was assessed using FastQC v. 0.11.9 (Babraham Bioinformatics, https://www.bioinformatics.babraham.ac.uk/projects/fastqc/) and adapters and low‐quality reads were trimmed using TrimGalore v. 0.6.1 (Krueger et al. 2023), which is a wrapper for CutAdapt (Martin 2011). We specified a quality threshold of 10, excluding very low‐quality bases, which is unlikely to negatively affect mapping and differential expression results (Williams et al. 2016). Following trimming, reads were again assessed with FastQC and MultiQC v.1.12 (Ewels et al. 2016). On average, trimmed files contained 25.3 +/− 0.8 M reads (Table S2). After sequencing and quality control, our study included six individuals from each treatment of CO flies and seven individuals from each treatment of CH flies (Table S1).

### 2.3 Read Mapping and Transcript Quantification

To quantify gene expression, trimmed RNAseq reads were aligned against the library of coding transcripts from our in‐house annotation (Supplementary Methods 2 in the Appendix S1; Figure S1) using Salmon v. 20180926 (Patro et al. 2017). We ran Salmon using default parameters, specifying FR strandedness (ISF flag) and the –gcBias flag, as insects tend to have lower GC content than many model systems. Transcript counts (transcripts per million) were concatenated into a single TXImport object for downstream gene‐level analyses in the statistical platform R v. 4.2.3 (R Core Team 2023) using an inhouse R script (available on GitHub https://github.com/rstewa03/Tconura_expressionPlasticity.git Steward 2025) in the Appendix S1, salmon2dds.R) and the packages GenomicFeatures v.1.52.2 (Lawrence et al. 2013), tximport v.1.28.0 (Soneson, Love, and Robinson 2016), supported by the tidyverse (Wickham et al. 2019). One of the samples (P18653_176, OO treatment) had very poor alignment and quantification rates and was excluded. Otherwise, the proportion of trimmed reads aligned and quantified against transcripts ranged between 30% and 55% and did not differ between CH and CO host races (Figure S2).

### 2.4 Differential Expression Analysis

To identify genes that were differentially expressed (DE) depending on host race and cross‐fostering, differential gene expression analysis was performed using the DESeq2 package v.1.36.0 (Love, Huber, and Anders 2014) in R. Transcript abundance estimates were converted into gene abundance estimates, which were prefiltered, keeping only those genes with at least five reads in a minimum of six samples, leaving 11,701 expressed genes. Read counts were normalised for visualisation and unsupervised clustering of gene expression using the ‘regularised log’ transformation implemented in DESeq2. To uncover major patterns of gene expression, a principal component (PC) analysis (stats::prcomp; v.4.2.3; R Core Team 2023) was performed on the 5000 genes with the most variable expression after normalisation, leaving out lowly expressed and invariable genes.

We tested for pairwise differential expression among treatments (Figure 1E). Specifically, we compared larvae sampled from their original host races (H vs. O), larvae cross‐fostered to their natal host plants (HH vs. OO) as well as comparisons of HH larvae to CH larvae cross‐fostered to *C. oleraceum* buds (HH vs. HO) and OO larvae to larvae cross‐fostered to *C. heterophyllum* buds (OO vs. OH) (see Table S3, Column A, for a full list of comparisons). We first fit models to the filtered set of genes with treatment as the predictor. We increased minReplicatesForReplace to 8 to make sure that outliers were refit with group averages for all contrasts, reducing the chance of false positives. We calculated log‐fold changes and assessed significant differences between treatment pairs by applying the adaptive Student's *t* prior shrinkage estimator (apeglm). We used the shrinkage estimator to calculate the *s*‐value (false sign rate, FSR; Stephens 2017) instead of the adjusted *p*‐value. Rather than evaluating whether the difference between two groups is zero, the FSR tests the probability that the sign of the effect is likely to be true (Stephens 2017). One motivation for using the *s*‐value for our data was that we found considerable within‐group variation in our samples. After adjusting log‐fold changes for this variability using the DESeq2 function lfcshrink, we still found highly significant adjusted *p*‐values for very small log‐fold changes (e.g., LFC = 0.001 or a change of 1.0007%). While very small changes in gene expression can have phenotypic effects, we preferred to focus on those genes for which we were confident in the sign of the effect. As recommended, we used a smaller significance threshold than generally used for an FDR. We found that a threshold of *s* < 0.001 identified similarly sized gene sets as an FDR of 0.05 (https://support.bioconductor.org/p/133091/; Stephens 2017; Zhu, Ibrahim, and Love 2019), but included values ranging from *s* < 0.01 in Table S3 to enable the reader to compare significance thresholds.

### 2.5 Weighted Gene Coexpression Network Analysis

To further explore host race‐specific gene expression and the extent to which the host races can plastically adjust gene expression when feeding on different hosts, we identified groups of genes, or modules, with similar expression across samples using a weighted gene coexpression network analysis with WGCNA v.1.72‐5 (Langfelder and Horvath 2008, 2012) on the regularised log‐transformed expression estimates. The soft thresholding power was selected as the first scale‐free topology fit index to exceed 0.8, using signed correlations (Langfelder and Horvath 2008). We specified a signed network using biweight midcorrelation (bicor), required a minimum module size of 30 and merged any modules that were over 70% related. The biweight midcorrelation is more robust to outliers than is a standard Pearson correlation (Langfelder and Horvath 2008). Both the minimum module size and the merge cut height were chosen to reduce the number of very small modules. A module membership threshold of 0.5 was used to exclude genes with a poor fit in any given module. Genes with higher membership in a given module are more likely to represent highly interconnected ‘hub’ genes within the module (Langfelder and Horvath 2008).

To uncover the factors affecting module expression, we correlated the standardised expression estimates, called eigengenes, for each individual in each module with five binary variables: host race, stress, reciprocal plasticity, CH plasticity and CO plasticity (Figure 3A, Table S2). Individuals were assigned 0 or 1 for each variable, for example, CH flies were assigned 1 for host race while CO flies were assigned 0, cross‐fostered flies were assigned 1 for stress while directly sampled flies were assigned 0, etc. (Figure 3A). We calculated Pearson's correlation coefficients and Student's asymptotic *p*‐values corrected for multiple comparisons across modules using the Benjamini–Hochberg method (*n* = 21 tests). We visualised these correlations across modules using ComplexHeatmap v. 2.12.1 (Gu 2022; Gu, Eils, and Schlesner 2016).

### 2.6 Functional Enrichment of Gene Sets

Functional enrichment of DE genes was performed with TopGO v.2.48.0; (Alexa and Rahnenfuhrer 2022) using a functional annotation made with EggNOG mapper v2 (Cantalapiedra et al. 2021; Huerta‐Cepas et al. 2019) (Supplementary Methods 2 in the Appendix S1). We required a minimum of 20 genes to perform gene set enrichment analysis (GSEA) and excluded gene ontology (GO) terms that were associated with fewer than five genes. GSEA tested for overrepresentation of GO terms using one‐sided Fisher's exact tests (parent–child algorithm). A threshold of *p* < 0.01 was set to identify significantly enriched terms describing biological processes and molecular functions.

### 2.7 Population Differentiation, Divergence and Signatures of Selection

We tested the extent to which expression differences between host races were associated with regions of genomic divergence, specifically a putative inversion segregating between the two host races (Steward et al. 2024). We used site allele frequency files from Steward et al. (2024) to calculate *F*_ST_ and *d*_XY_ between the CH and CO source populations and to estimate nucleotide diversity (*π*) and Tajima's *D* over 50 kb nonoverlapping windows throughout the 1.9G *T. conura* genome. The two‐dimensional folded site frequency spectrum (SFS) was used to calculate *F*_ST_ using the Bhatia estimator (ANGSD v. 0.940, realSFS fst index; realSFS fst stats2; Korneliussen, Albrechtsen, and Nielsen 2014). To calculate *d*_XY_, we first recalculated the 2D‐SFS for every 50 kb window, then calculated *d*_XY_ using a modified version of dxy_wsfs.py script from D. Marques (https://github.com/marqueda/PopGenCode/blob/master/dxy_wsfs.py, accessed on Nov. 2023) modified to run in R (dxy_wsfs.R; available on GitHub https://github.com/rstewa03/Tconura_expressionPlasticity.git Steward 2025). We also used ANGSD to calculate *π* and Tajima's *D* over 50 kb windows. We calculated the difference in nucleotide diversity (Δ*π*) and Tajima's *D* (Δ*D*) as *π*_CH_—*π*_CO_ and *D*_CH_ ‐*D*_CO_ respectively. Outlier windows for each metric were identified as windows more than 3× the standard deviation above (*F*_ST_, *d*_XY_, Δ*π* and Δ*D*) or below (Δ*π* and Δ*D*) the mean.

We intersected gene loci (+/−2 kb) with 50 kb windows and used hypergeometric tests in R (stats::phyper) to evaluate whether DE genes were more likely to appear in highly differentiated windows than expected by chance. We further tested whether the relationship between expression and genomic divergence differed inside and outside of the putative inversion using Kruskall–Wallis and Dunn's multiple‐comparison tests with rstatix v.0.7.2 (Kassambara 2021). Although we did not calculate population genomic metrics for individual genes, we nevertheless ran a second set of tests as a control, using a subset of non‐DE genes matched for length with the DE genes generated with nullranges, v.1.2.0 (Love et al. 2022), as gene length covaries with some population genomic metrics.

## 3 Results

Using unsupervised clustering approaches, we determined host race was the main driver of gene expression differences among both directly sampled and cross‐fostered *T. conura* larvae. The CH and CO host races were separated on the first and second axes in a PC analysis of the 5000 genes with the most variable expression (Figure 1F). In contrast, larvae did not cluster by cross‐fostering treatment on any of the first five PC axes (Figure S3). Consistently, hierarchical clustering grouped larvae by host race, especially CO larvae, with some evidence that cross‐fostered larvae tended to cluster together within the CO host race regardless of whether they were moved to the natal or novel host (Figure S4). Jointly, these expression patterns suggest that constitutive expression differences affect a much larger portion of the transcriptome than plastic responses to the cross‐fostering treatment.

### 3.1 Differential Expression Between Host Races

**FIGURE 2:**
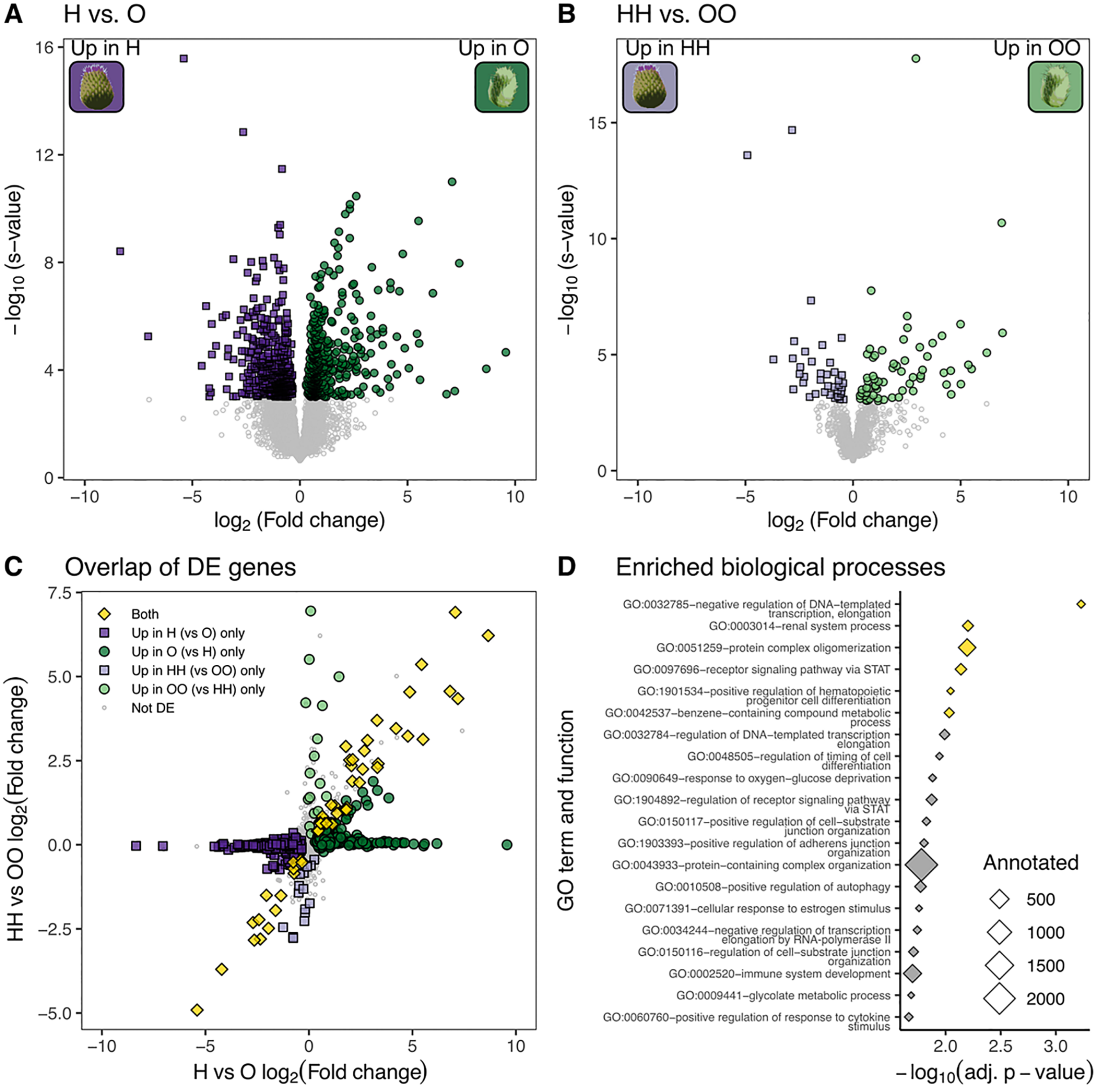
Differential expression between CH and CO larvae. Differentially expressed (DE) genes between (A) control lines (H vs. O) and (B) larvae cross‐fostered to their natal host (HH vs. OO). Differential expression was tested using Wald's Chi‐squared tests and significantly DE genes had an *s*‐value < 0.001. (C) Genes that are significantly DE in both A and B (yellow). (D) Biological processes enriched (adjusted *p* < 0.01, Fisher's exact test adjusted with the parent–child algorithm, yellow) in the subset of overlapping genes in A and B; see Tables S4 and S5 for a detailed list of enriched GO terms.

Larvae of the CH and CO host races use different genes to deal with toxic or harmful components in their host plants. Comparing the control lines H and O, we detected 488 genes more highly expressed in H larvae and 445 genes more highly expressed in O larvae, together making up 7.97% of the filtered, expressed transcriptome (Figure 2A; Table S3). Both gene sets were primarily enriched for GO terms involved in regulation of biological processes and metabolism (an overview of enriched GO terms is available in Tables S4 and S5). Detoxification (GO:0098754, *p* = 7.6 × 10^−5^), cellular detoxification (GO:1990748, *p* = 3.1 × 10^−5^) and cellular response to toxic substance (GO:0097237, *p* = 3.5 × 10^−3^) were all explicitly enriched in the genes upregulated in O larvae. These genes were also enriched for biosynthesis and metabolism of glycoproteins (Table S4), which are involved in herbivorous insect responses to plant chemical defences like flavonoids (Aurade et al. 2011; Dermauw and Van Leeuwen 2014), the dominant chemical defence in thistles (Jordon‐Thaden and Louda 2003). Genes upregulated in O larvae were also enriched for protein folding (GO:0006457, *p* = 9.1 × 10^−5^) and the endoplasmic reticulum unfolded protein response (GO:0034975, *p* = 6.0 × 10^−3^), which are characteristic of the insect‐integrated stress response (Rosche et al. 2021). Genes upregulated in H larvae were highly enriched for various metabolic processes (e.g., GO:0080090, *p* = 3.2 × 10^−11^), and marginally enriched for toxic responses, such as toxin transport (GO:1901998, *p* = 3.3 × 10^−2^), and response to chemical stimulus (GO:0070887; *p* = 2.3 × 10^−3^) and chemical stress (GO:0062197; *p* = 4.7 × 10^−2^). Together, these DE genes confirm that *C. oleraceum* and *C. heterophyllum* represent distinct chemical and nutritional environments and that CH and CO larvae have different molecular mechanisms that enable feeding on their host plants.

In comparison, many fewer genes were DE between larvae of the two host races when cross‐fostered to their natal hosts (HH vs. OO; Figure 2B; all DE genes are reported in Table S3). This was largely due to high variation within each of the sample groups, likely introduced by the stress of cross‐fostering. Overall, 40 genes were more highly expressed in HH larvae, and 64 genes were more highly expressed in OO larvae. Only 53 of these genes overlapped with the 933 genes that were DE between control larvae (Figure 2C). These 53 genes were enriched for a small set of functions that reveal the consistent differences between CH and CO specialist larvae feeding on their natal hosts (Figure 2D, Table S5). Renal system processes were enriched (GO:0003014, rank = 2, *p*‐value = 6.3 × 10^−3^), which in dipterans implicates structures like nephrocytes and Malpighian tubules that are involved in fluid, electrolyte and pH balance, and disposal of toxic waste products (Denholm and Skaer 2009; Xu et al. 2022). Importantly, overlapping DE genes were also enriched for metabolism of benzene‐containing compounds (GO:0042537, rank = 6, *p*‐value = 6.0 × 10^−3^). Flavonoids are composed of two benzene rings linked by a three‐carbon pyran ring (Dias, Pinto, and Silva 2021), so enrichment of this term may indicate that defensive flavonoid chemical profiles of *C. heterophyllum* and *C. oleraceum* affect expression and performance of specialist larvae on these host plants. However, it is unclear how divergent these chemical profiles are as the nonvolatile chemical landscapes of the buds have not been investigated in a comparative framework.

### 3.2 Expression Plasticity in Ancestral and Derived Host Races

We tested to which extent larvae can plastically shift gene expression in response to a different host, and whether this ability differs between the ancestral (CH) and derived (CO) host races. Overall, larvae cross‐fostered to a novel host had very few DE genes compared to larvae cross‐fostered to their natal host, with fewer than 10 genes DE in ancestral (HH vs. HO) and derived (OO vs. OH) larvae (Figure S5A,B). Nevertheless, even a small number of DE genes could represent adaptive transcriptional plasticity. We predicted that genes with adaptive plastic expression on novel hosts would match the expression differences found between the two host races. For example, if gene expression is adaptively plastic, differences between HH and HO larvae should match those between HH and OO larvae, that is, expression differences should affect the same genes, and the differences in expression should be in the same direction. However, we found only a single gene matching this pattern for each of the host races (Figure S5C,D). In the ancestral CH host race, this gene was Tcon_g9682, aka RARS, which belongs to the Class I aminoacyl‐tRNA synthetase family. Expression of RARS was elevated in HO larvae and all CO host race larvae (Figure S5C inset). In the derived CO host race, Tcon_g14248 (TRAPCC5, a trafficking protein particle complex) matched the expected pattern of expression based on host plant rather than host race. However, when we further investigated TRAPPC5 expression across treatments, we found that this gene was not in the subset of shared DE genes between host races (Figure 2C). Rather, control H larvae and cross‐fostered HH larvae had very different expression levels of TRAPPC5, as did O and OO larvae (Figure S5D inset), making this a poor candidate for host shift‐induced transcriptional plasticity. These results could suggest that neither host race has the capacity for extensive, adaptive transcriptional plasticity when switched to a novel host during the third instar.

### 3.3 Host Race‐, Stress‐ and Plasticity‐Associated Patterns in Coexpressed Genes

**FIGURE 3:**
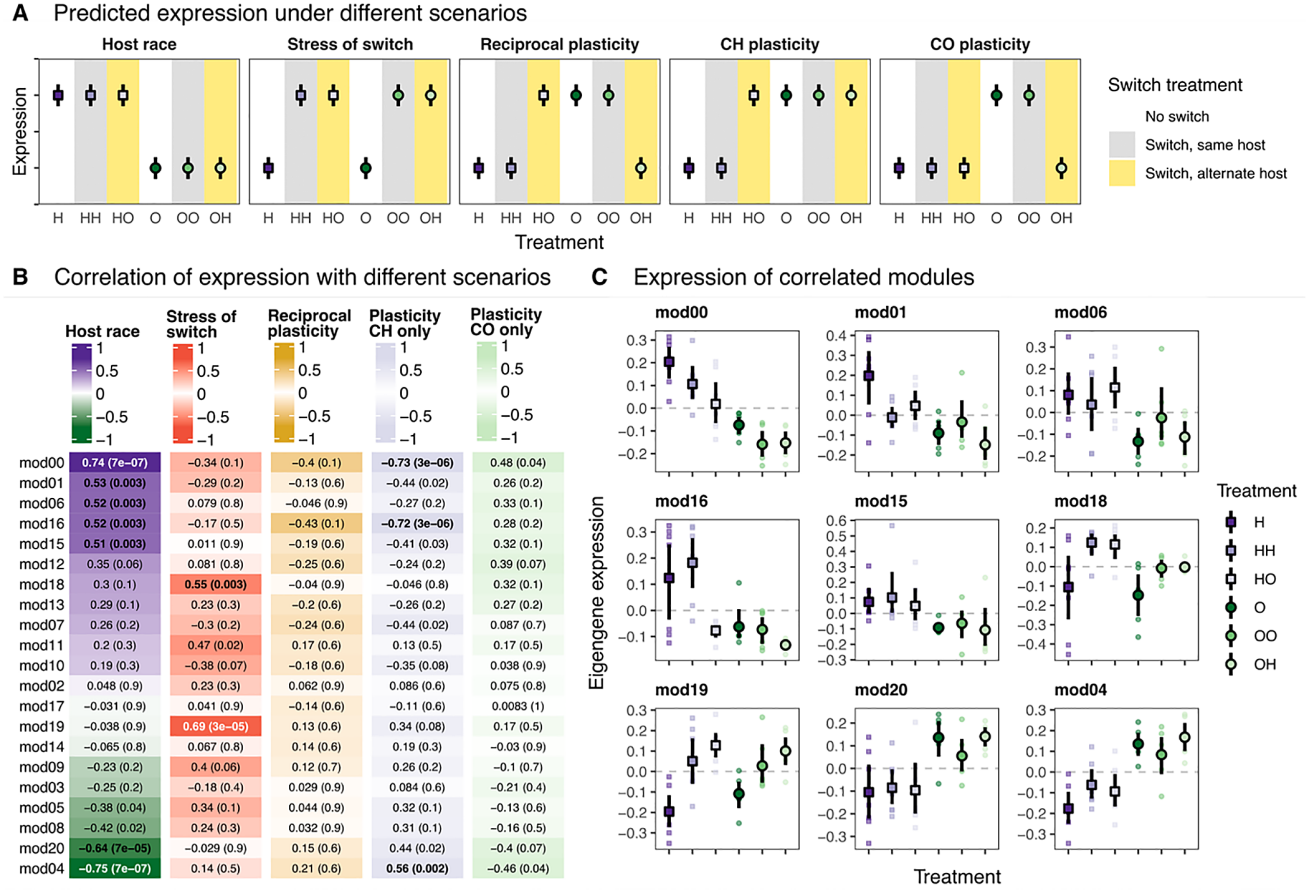
Correlation of weighted gene coexpression networks with expected patterns of expression across treatments. (A) Predicted patterns of gene expression under different scenarios. Coloured backgrounds correspond to treatment: flies were sampled from their original buds (white) or switched to another host plant of the native (grey) or alternate (yellow) host plant. Colours and symbols correspond to the treatments; see legend for Panel C. (B) Heatmaps showing correlation coefficient ranging between −1 and 1 of module expression for each eigengene with dummy variables representing different scenarios: Host race (1 = CH, 0 = CO), stress of cross‐fostering (1 = switched, 0 = not switched), reciprocal plasticity (1 = feeding on *C. oleraceum*, 0 = feeding on *C. heterophyllum* ), plasticity in CH only (1 = CO host race or HO treatment, 0 = CH feeding on *C. heterophyllum* ) and plasticity in CO only (1 = CH host race or OH treatment, 0 = CO feeding on *C. oleraceum*). Pearson's correlation coefficient and adjusted student asymptotic *p*‐value are in bold if they meet our threshold for a meaningful, significant correlation with the predicted patterns in Figure 3A (*r* > 0.5 and *p* < 0.01). (C) Expression of eigengenes (first principal component of gene expression) across treatments for modules with significant correlations in (B). Small points show eigengene expression for each sample, and large points and black vertical lines represent means and 95% confidence intervals respectively.

As an alternative test of constitutive and plastic differences in gene expression across treatments, we used weighted gene coexpression network analysis to identify and compare the expression of modules of coexpressed genes with predicted expression patterns (Figure 3A). We uncovered 20 modules of coexpressed genes (mod00 comprises unclustered genes; Figure 3B, Figure S6A; Table S6). We tested if these modules were meaningfully correlated (*r* > 0.5) and significantly associated (adj. *p* < 0.01) with predicted expression patterns (Figure 3A).

We found that expression in one‐third of the modules was correlated with larval host race (Figure S6B, Figure 3B). None of the modules were expressed as expected under reciprocal plasticity, where larval expression reflects the host plant they were feeding on regardless of host race. Similarly, no modules were expressed in a manner consistent with CO plasticity, where CO larvae cross‐fostered to *C. heterophyllum* have CH‐like expression. However, three modules had a significant signature of CH plasticity, with CH larvae cross‐fostered to *C. oleraceum* having CO‐like expression.

Of the modules associated with CH plasticity, mod16 (*n* = 76 genes) was most strongly correlated (*r* = −0.72, *p* = 3 × 10^−6^). Genes in mod16 had low expression in CO larvae (O, OO and OH) and CH larvae feeding on *C. oleraceum* (HO), and highly variable expression in H and HH larvae (Figure 3C, Figure S6). This module was overwhelmingly enriched for reproductive processes (Figures S7D and S8D), suggesting CO larvae may have a delayed life history progression relative to CH larvae of the same size. CH larvae thus delay the onset of reproductive development plastically when growing on *C. oleraceum*, either directly or indirectly (e.g., by slowing growth).

Despite comprising genes not specifically assigned to a coexpression module, mod00 (*n* = 3059 genes) expression was also significantly correlated with CH plasticity (*r* = −0.73, *p* = 3 × 10^−6^). It was also the module most correlated with the CH host race overall (*r* = 0.74, *p* = 7 × 10^−7^). The main pattern for mod00 was that its expression generally was lower in the CO than in the CH host race, but cross‐fostered larvae from the CH host race also tended to express lower levels of these genes. This module was enriched for cell cycle processes, such as centriole assembly (GO:0098534, rank = 1, *p* = 5.2 × 10^−4^) and replication (GO:0007099, rank = 2, *p* = 6.2 × 10^−4^), chromatin (GO:0006325, rank = 3, *p* = 1.4 × 10^−3^) and microtubule organising centre (GO:0031023; rank = 6, *p* = 2.7 × 10^−3^) organisation (Figures S7A and S8A). Furthermore, this module was enriched for salivary gland histolysis (GO:0035070, rank = 8, *p* = 5.0 × 10^−3^), a specific process of salivary gland breakdown during dipteran metamorphosis (de Cassia Santos Przepiura et al. 2020). Together with mod16, this may support a faster life history in CH flies that is slowed by cross‐fostering in *C. oleraceum* buds.

Finally, mod04 (*n* = 1185 genes) was the third module correlated with CH plasticity (*r* = 0.56, *p* = 0.002; Figure 3B). However, expression of this module did not fully match our expectations for CH plasticity when assessed visually (i.e., Figure 3A), and overall, the module was also highly correlated with host race (*r* = −0.75, *p* = 7 × 10^−7^), which likely influenced the correlation with CH plasticity. However, mod04 contains RARS (Tcon_9682), which we previously identified as plastically expressed in HO flies using our differential expression approach. The module was also enriched for numerous processes involved in cellular respiration, detoxification and protein transport (Figures S7 and S8).

Two modules were significantly correlated with a stress effect from cross‐fostering: mod19 (*n* = 40 genes, *r* = 0.69, *p* = 3 × 10^−5^) and mod18 (*n* = 49 genes, *r* = 0.55, *p* = 0.003). In these modules, expression in the cross‐fostered larvae was consistently higher than in control larvae. This stress effect was corroborated by significant functional enrichment in mod19 of regulation of metabolic and catabolic processes (GO:0019222, rank = 3, *p* = 2.9 × 10^−3^; GO0031323, rank = 7, *p* = 5.3 × 10^−3^; GO:0031329, rank = 1, *p* = 2.2 × 10^−3^; GO:0009894, rank = 4, *p* = 3.8 × 10^−3^; Figure S7F) and cytokine‐like and interferon signalling and response (GO:0060338, rank = 5, *p* = 4.2 × 10^−3^; GO:0060759, rank = 30, *p* = 2.2 × 10^−2^), which are associated with immune system responses (Labropoulou et al. 2024; Rosche et al. 2021). Mod18 was enriched for midgut development (GO:0055123, GO:0007496 and GO:0007494) and endothelial cell development and regeneration (Figure S7G). The functional enrichments of these modules suggest that the process of moving to a new host is metabolically and immunologically stressful, regardless of whether the new host is the natal, adapted host or the novel, challenging host.

### 3.4 Expression Differences Associated With Genomic Architecture and Population Divergence

**FIGURE 4:**
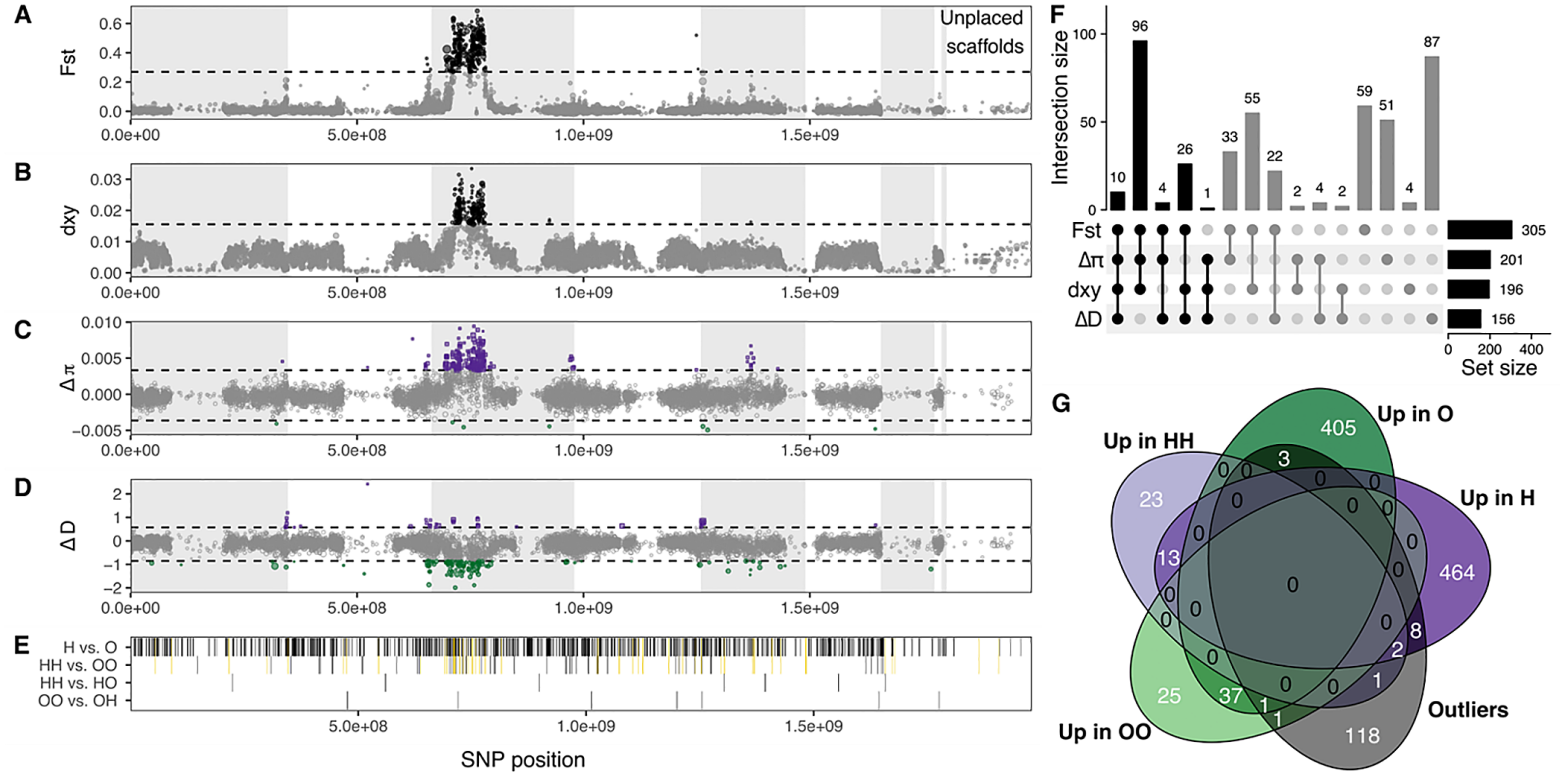
Overlap between DE genes and regions of high genomic differentiation and divergence between CH and CO populations. (A) *F*_ST_ (Bhatia estimator) and (B) *d*_XY_ over 50 kb windows between the CO and CH populations used for the cross‐fostering experiments (Figure 1E). Horizontal dashed lines represent mean + 3 standard deviations. Outliers are shown in black. (C) Nucleotide diversity difference (Δ*π*) and (D) Tajima's *D* difference (Δ*D*) between CH and CO populations. Horizontal dashed lines represent the mean +/−3 standard deviations, and outliers are shown in purple if *π* or *D* were higher in CH and green if higher in CO. Windows with < 20% coverage were excluded, resulting in gaps along the contigs, which are ordered according to putative linkage groups (delineated as light grey or white bands, with the rightmost white band containing unscaffolded contigs). (E) Genes that were DE between treatments were distributed throughout the genome. Yellow bands show genes that overlap between H versus O and HH versus OO (Figure 2C, yellow diamonds). (F) UpsetR plot of genes falling in outlier windows for each metric. Horizontal bars show total genes identified for each metric, while vertical bars show the size of the overlap between gene sets. We classified outlier genes as those overlapping outlier windows in three of four population genomic metrics (black bars). (G) Venn diagram visualising overlap between DE genes (H vs. O and HH vs. OO) and outlier genes.

To uncover the relationship between expression differences and genomic architecture, we assessed distribution of DE genes in relation to genetic divergence. Steward et al. (2024) identified a large putative inversion (~104 Mb, 89 contigs) on LG3 in the *T. conura* assembly as the major locus of genomic divergence between the host races in parallel contact zones east and west of the Baltic Sea. Here, we focus only on the two source populations for our cross‐fostered larvae (Figure 1B) to test whether constitutive or plastic expression differences tended to be associated with highly differentiated regions inside and outside of this inversion (Figure 4). As expected, we detected a large peak at the putative inversion in both *F*_ST_ and *d*_XY_, which quantify genomic differentiation and absolute nucleotide divergence, respectively (Figure 4A,B). This region is also characterised by a positive peak in Δ*π* (nucleotide diversity higher in the CH host race than the CO host race, Figure S9) and a negative Δ*D* (Tajima's *D* lower in the CH host race than the CO host race, where Tajima's *D* is an estimate of how *π* differs from neutral expectations and a rough test for selection; Figure S9; Figure 4C,D). Using the two source populations, we identified a subset of genomic windows that were consistently supported by differentiation, divergence and selection metrics (> mean ± 3 std. deviations in three of four metrics). These windows contained 134 genes (Figure 4F) and were overwhelmingly located within the putative inversion. Only one gene, Tcon_g8259 (unnamed in our functional annotation, possible ortholog of Dmel\CG13928, blastp score = 63.1 and *e*‐value = 3.7 × 10^−10^), fell in an outlier window outside of the inversion.

We next tested whether DE genes between host races were also likely to overlap these regions of high differentiation and divergence. Genes that were DE between H and O, between HH and OO and the subset of genes found in both gene sets (i.e., ‘both’ in Figure 2C, *n* = 53, yellow lines in Figure 4E) overlapped with outlier genes significantly more than expected by chance (H vs. O: 14 genes, odds ratio = 3.06, hypergeom. test FDR = 3.30 × 10^−4^; HH vs. OO: 5 genes, odds ratio = 9.87, hypergeom. test FDR = 6.53 × 10^−5^; ‘both’: 3 genes, odds ratio = 11.45, hypergeom. test FDR = 3.67 × 10^−4^; Tables S7 and S8). These patterns became stronger when we limited the set of outlier genes to those that were also expressed (92 out of 134 genes). We also found a significant enrichment of DE genes falling within the putative inversion, regardless of whether they overlapped outlier windows, both when testing all inversion genes (1343 genes) and those that were expressed (777 genes; Table S8). Gene density within the inversion (1.29 × 10^−05^ genes/bp) is only marginally higher than the genome‐wide average (1.27 × 10^−05^ genes/bp) and is unlikely to explain enrichment of DE genes in this region. None of the genes that were DE between HH and HO or between OO and OH overlapped with outlier windows (Figure 4E; Table S8), and only one DE gene between OO and OH fell within the putative inversion.

We investigated if there was evidence for increased differentiation, divergence or selection for DE genes by assessing differences in *F*_ST_, *d*_XY_, Δ*π* and Δ*D*, *π* or Tajima's *D* between genes that were or were not DE. This analysis was performed separately for genes inside and outside of the inversion. Overall, genes within the inversion that were DE between host races (H vs. O) were not significantly different from genes that were not DE, apart from marginally significantly lower nucleotide diversity in the CO population (Figure S10; Table S9). However, among the genes outside of the inversion, DE genes were less divergent (lower *d*_XY_; Figure S10B) and accordingly had significantly lower *π* in both populations (Figure S10E,F). This pattern was maintained when we subset the non‐DE genes to match the gene length distribution of the DE genes and may result from purifying or negative selection on or near genes that are functionally important for host plant use. No significant differences were found when we focused on genes that were DE in HH versus OO (Figure S11; Table S10).

Finally, we compared these metrics among the expression modules (Figure S12). While there were some overall patterns (e.g., *d*_XY_ and *π* in both populations tended to be higher in mod03, mod06, mod07 and mod09, and lower in mod04, mod05, mod13 and mod19, Dunn's multiple comparison, Table S11), these patterns did not align with module correlations with host race, stress or plasticity (Figure 3B, Figure S12).

## 4 Discussion

To what extent evolution of gene expression enables adaptation to novel environments, and how altered gene expression interacts with genetic divergence and genomic architecture during this process is crucial for understanding the adaptive potential of organisms (Ballinger et al. 2023; López‐Maury, Marguerat, and Bähler 2008; Pavey et al. 2010; Triant, Nowick, and Shelest 2021). Importantly, whether the changes in gene expression that enable the use of novel environments are constitutive or plastic has consequences on whether an organism retains the ability to use ancestral environments (Celorio‐Mancera et al. 2023; Ho et al. 2020; Ho and Zhang 2018). Few studies have addressed whether the process of adaptation to a novel niche affects the capacity for transcriptional plasticity and subsequent broad niche use.

Here, we document constitutive and plastic changes in gene expression underlying adaptation to a novel host plant in the peacock fly, *T. conura* . Overall, we found limited evidence for adaptive transcriptional plasticity in response to feeding on the alternate host in either the ancestral CH host race or the derived CO host race. Nevertheless, coexpression analysis uncovered three modules of genes for which expression in CH larvae feeding on *C. oleraceum* shifted to match expression of the CO host race, providing some evidence for the hypothesis that transcriptional plasticity would be greater in the ancestral than the derived host race. In contrast, we found extensive constitutive transcriptional differences between the two host races, several of which were robust to the stressful cross‐fostering treatment. Genes that were DE in the two host races were enriched for metabolism and detoxification functions, likely reflecting adaptations to differences in the nutritional and chemical profiles of the thistle host plants. Finally, we found that these constitutively DE genes were more likely than expected by chance to fall in highly divergent regions of the genome, specifically within the large putative inversion on LG3. Together these results suggest that gene expression evolution has contributed considerably to adaptive phenotypic divergence between CH and CO lineages and that genomic architecture is playing an important role in shaping the transcriptomes of these host races.

### 4.1 Gene Expression Divergence Between Host Races

Host plant chemistry is a major driver of host plant specialisation and diversification in insects (Birnbaum and Abbot 2020; Jaenike 1990; Jousselin and Elias 2019; van der Linden, WallisDeVries, and Simon 2021). Our results support a critical role for thistle chemistry in the adaptive divergence of gene expression between the CH and CO host races of *T. conura* . Broadly, we found that DE genes between the host races are significantly enriched for detoxification and responses to toxic chemicals, suggesting that the host races rely on different molecular mechanisms to confront the challenges of feeding on their adapted hosts. Specifically, genes that were DE both between the control H and O treatments and between HH and OO were enriched for metabolism of benzene‐containing compounds, which may include flavonoids (Dias, Pinto, and Silva 2021). Flavonoids, the dominant defensive class in thistles (Jordon‐Thaden and Louda 2003), can have deterrent or even toxic effects on herbivorous insects, but can also act as oviposition attractants or feeding stimulants (Aurade et al. 2011; Dias, Pinto, and Silva 2021; Mierziak, Kostyn, and Kulma 2014). Functional enrichment of metabolism of benzene‐containing compounds suggests the flavonoid chemical profiles of *C. heterophyllum* and *C. oleraceum* are important selection pressures acting on gene expression in specialist larvae on these host plants.

Constitutive differences in gene expression between the CH and CO host races likely act as barriers to gene flow. Larval feeding assays have shown that both host races have significantly reduced survival on the wrong host plant (Diegisser, Johannesen, and Seitz 2008). Our results suggest that constitutive differences in gene expression, adapted to the different chemical and possibly nutritional profiles of the host plants, are part of the molecular mechanisms underlying these fitness costs. Limited plasticity, especially in the derived CO host race, could mean that larvae are not buffered against maladaptive oviposition decisions of adult females, which may result in strong selection against laying eggs in the wrong host plants in parapatric and sympatric contact zones. However, it is possible that larvae exhibit plasticity early in development that is gradually lost over longer exposure to the host environment. This could imply that the full potential for plasticity is not captured by the current design and is discussed in more detail below.

We expect divergence in both *cis‐* and *trans‐* regulatory elements to contribute to the extensive expression differences uncovered between the host races. For example, many of the genes found to be both DE and highly genetically divergent between host races were annotated with regulatory roles, including transcription factors and methyltransferases involved in epigenetic modifications for active gene transcription (Table S7). Divergence in evolved regulatory networks has the potential to lead to transgressive phenotypes in hybrids that, while not intrinsically inviable in the classic sense of Batesian–Dobzhansky–Muller incompatibilities, may in turn cause reduced fitness in available niches (Thompson et al. 2023). While we have yet to explore gene expression and fitness in F1 hybrids or subsequent backcrosses, hybridisation could produce combinations of *cis‐* and *trans‐* regulatory elements that disrupt the host‐specific gene expression profiles we have uncovered in this study, potentially leading to reduced hybrid fitness.

### 4.2 Genomic Architecture of Differential Gene Expression

Our results suggest an important role for the large inversion of LG3 identified by Steward et al. (2024) in gene expression divergence, as the inverted region was significantly enriched for DE genes. Inversions are well known to be important drivers of ecological divergence and speciation (Berdan et al. 2023; Faria et al. 2019; Feder and Nosil 2009; Fuller et al. 2016; Westram et al. 2022) and have been identified in a number of systems exhibiting rapid local adaptation to and divergence in novel niches (Ayala, Guerrero, and Kirkpatrick 2013; Koch et al. 2021; Kollar et al. 2023; Lee et al. 2017; Lowry and Willis 2010; Morales et al. 2019; Twyford and Friedman 2015). In particular, inversions have been found to underlie ecological divergence and reproductive isolation in several well‐known examples of host plant‐associated differentiation and host race formation. Inversions segregate between apple‐ and hawthorn‐infesting populations of another Tephritid fly, *Rhagoletis pomonella* (Egan et al. 2015; Feder and Nosil 2009; Ragland et al. 2017). Large inversions also underlie both a host‐associated colour polymorphism (Nosil et al. 2018) and inter‐ and intraspecific variation in feeding on redwood trees by *Timema* walking sticks (Nosil et al. 2023). However, experimental evidence for how inversions alter gene expression during local adaptation and divergence is still limited (Berdan et al. 2023).

Inversions can influence gene expression in two main ways (Berdan et al. 2023). First, inversion breakpoints can break genes or rearrange the structural relationships between regulatory elements and the protein‐coding regions of genes. Although this should only impact a small fraction of genes, depending on their roles within larger gene regulatory networks, genes at or near breakpoints could have cascading effects on gene expression throughout the genome. Second, as barriers to recombination in heterokaryotypic individuals, inversions can capture and accumulate divergent sequence variation more quickly than collinear regions of the genome (Faria et al. 2019; Schaal, Haller, and Lotterhos 2022), resulting in rapid evolution of both *cis‐*regulatory elements and coding regions of *trans‐*acting factors located within the inversion. For example, 80.6% of genes that were DE between inversion genotypes of the seaweed fly *Coelopa frigida* were located within the inversion (Berdan et al. 2021), suggesting a major role for *cis‐*regulatory sequence divergence within the inversion. Which of these mechanisms is more likely to be happening in the *T. conura* system is still unclear. We are currently unable to assess breakpoint dynamics due to lack of contiguity in our *T. conura* assembly. However, we have extensive evidence for sequence divergence between the inversion haplotypes resulting from reduced introgression and positive selection, especially in the CO host race (Steward et al. 2024), with the strong signals of selection in particular in the derived host race, suggesting a direct role in the adaptation to the different host plants. Moreover, windows containing DE genes tended to have lower nucleotide diversity in both populations regardless of whether they were found inside the inversion or not. Nevertheless, we did not find differences in *F*_ST_, *d*_XY_ or Tajima's *D* in windows around DE genes compared to those that were not DE. Thus, while we hypothesise that sequence divergence within the inversion is contributing to the large number of DE genes between the host races, we were unable to identify specific signatures of divergence or selection supporting this hypothesis. It is also unclear how introgression in other parts of the genome may affect the relative importance of DE genes within the inversion. Due to sampling restrictions and caution about the extent of gene flow in sympatry, CH flies were sampled in the allopatric host range, whereas CO flies were sampled in the sympatric host range (Figure 1B). We have since determined that the sympatric populations in southern Sweden appear to be the most differentiated (Steward et al. 2024). Nevertheless, there is still considerable introgression outside of the inversion (Steward et al. 2024) which may alter the extent of expression differences in these populations. Future research identifying the mechanistic basis of altered expression and investigating whether changes to regulatory regions, chromatin structure or topologically associating domains are responsible for the expression differences of genes both in and outside of the inversion, would improve the understanding of the ecological importance of inversions in different geographic modes of speciation.

### 4.3 Limited, Asymmetric Expression Plasticity

We expected transcriptional plasticity in response to alternative host plants (Celorio‐Mancera et al. 2023; Gibert 2017). We also predicted that strong selection on the CO host race when colonising and adapting to *C. oleraceum* should have led to genetic assimilation, that is, an evolved loss of expression plasticity, resulting in asymmetrically more plasticity in the ancestral CH host race. While overall evidence for transcriptional plasticity was limited, our results broadly fit the predictions under genetic assimilation. Specifically, three gene coexpression modules exhibited patterns of gene expression plasticity unique to CH larvae, while no modules exhibited patterns consistent with plasticity unique to CO larvae. However, these modules do not mainly contain genes involved in processes that are likely to provide adaptive advantages when metabolising the CO host plant. Thus, it remains unclear whether the asymmetric plasticity observed in mod00, mod04 and mod16 is adaptive, neutral or even nonadaptive. In order for plasticity to be adaptive, it should contribute to increased fitness in the new environment, and expression should match, or approach, the optimum for that environment (Ghalambor et al. 2015). And yet, expression matching may not always be adaptive. For example, mod16 genes, which were found to be enriched for reproductive development and maturation functions, were only expressed in noticeable amounts in H and HH larvae and were negligibly expressed in all CO larvae as well as CH larvae feeding on *C. oleraceum* (HO). All larvae in our study were visually size matched, but it is possible that reproductive development starts at a smaller size in CH than in CO larvae, and rather than matching an adaptive expression optimum in CO larvae, the reduced expression of development and maturation function of larvae in the HO treatment experienced delayed or stunted development by feeding on *C. oleraceum* even for a short period of time. Under this scenario, the ‘CH‐plasticity’ of mod16 genes may represent nonadaptive changes in gene expression, potentially reflecting delayed maturation from the stress of using a new host plant that may match CO larval expression by chance rather than improving performance on *C. oleraceum*. Similarly, mod00 genes were enriched for developmental functions, such as cell cycle regulation and salivary histolysis, both of which could be associated with prepupal development and could have been inhibited by feeding on a suboptimal host plant.

We did not find any evidence for reciprocal plasticity, in which both host races share host plant‐specific transcriptional patterns. One prediction of the oscillation hypothesis is that shared molecular mechanisms facilitate colonisation and recolonisation of novel and ancestral host plants (Celorio‐Mancera et al. 2023; Ho et al. 2020). Accordingly, Celorio‐Mancera et al. (2023) found that coexpressed gene modules were shared between generalist and specialist species of *Polygonia* and *Nymphalis* butterflies and similarly expressed when feeding on the same host plant. Even butterflies with the derived host repertoire were able to plastically express the same suites of genes across a range of host plants. In contrast to these findings, we do not find evidence for any shared ancestral mechanisms enabling reciprocal plasticity as predicted under the oscillation hypothesis. Such adaptive plasticity would be reflected in gene expression activated depending on host plant use in the CH and CO *T. conura* host races. Instead, the focal *T. conura* populations are highly specialised. As there are fitness costs associated with being raised on the alternative host plant (Diegisser, Johannesen, and Seitz 2008), full reciprocal plasticity may not be expected. Thus, the constitutive expression differences between the host races potentially reflect that genetic assimilation has taken place during the 0.5–1 million years they have evolved separately (Steward et al. 2024). The resulting limited plasticity in expression, in particular in the derived CO host race, could possibly reduce the survival prospects in face of environmental change altering the range of thistle species available. However, both host races currently have populations using multiple host plants within their distribution. CH flies oviposit and successfully develop in both *C. heterophyllum* and *C. palustre* in the northern British Isles (Diegisser et al. 2009; Romstöck‐Völkl 1997), and CO flies oviposit in *C. oleraceum* and *C. acaulon*, among others, in several regions of the Alps (Romstöck‐Völkl 1997). Transcriptional plasticity is known to be important for single populations using multiple hosts (Birnbaum and Abbot 2020), depending on the chemical similarity of the plants (Celorio‐Mancera et al. 2016). Whether these oligophagous populations would show greater transcriptional plasticity when exposed to a novel host would be a useful next step for understanding the roles of adaptive plastic phenotypes and their role in host plant shifts.

The importance of transcriptional plasticity in host use is a difficult question to address because larval age, experience and length of exposure can all interact to affect larval gene expression (Birnbaum and Abbot 2020; Schneider et al. 2024). The cross‐fostering design clearly introduced more variation in expression within treatment groups, as we found nearly an order of magnitude fewer genes DE between larvae cross‐fostered to their natal host (HH vs. OO) compared to larvae that were directly sampled (H vs. O). Thus, our design was likely only able to reveal genes with the strongest or most tightly regulated differences in expression between cross‐fostered larvae, and larger sample sizes in future studies will help overcome the statistical consequences of this within‐group variation. Another potential concern of our study design is that larvae were only allowed to feed on the novel host plant for 6 h, which may not be long enough to detect a consistent, regulated change in gene expression on the new host. For example, Schneider et al. (2024) found that after 2 h of feeding on a new host, gene expression in larvae of the butterfly *Polygonia c‐album* was still best explained by the natal host. It was only after 17 h that the main effect of the second host plant on gene expression could be detected. Despite this concern, our results clearly show that *T. conura* larvae of both host races can rapidly alter gene expression in the 6‐h timeframe. We detected two small coexpressed modules that were consistently upregulated in response to the acute stress of the cross‐fostering design. While stress response may be faster than adaptive plastic responses, this shows the potential for a transcriptional response within the experimental time frame. The upregulated coexpressed modules were strongly enriched for functions involved in the insect‐integrated stress response (Harding et al. 2003; Rosche et al. 2021). For future studies, quantifying phenotypic consequences of cross‐fostering in the form of, for example, differences in survival, development time or weight gain depending on the new host plant could help differentiate stress responses from adaptive plasticity. It is also unclear how transcriptional plasticity changes over the lifespan of a developing insect, and how this transcriptional plasticity translates to phenotypic plasticity and fitness. Potentially, larvae exhibit plasticity early in development which is gradually lost over longer exposure to the host environment, which could imply that the full potential for plasticity is not captured by the current study design. To our knowledge, there have been no studies on how host‐associated transcriptional plasticity changes through juvenile and adult development (but see Celorio‐Mancera et al. 2013). However, phenotypic assays suggest that holometabolous larvae often habituate to their natal feeding environment, altering subsequent acceptance (Huang and Renwick 1995) or performance (Söderlind, Janz, and Nylin 2012). Additional research would be needed to uncover how such changes in phenotypic plasticity across life stages could relate to underlying transcriptional plasticity and is an interesting avenue for future research.

In conclusion, extensive constitutive differences in gene expression between the host races, especially for genes likely involved in processing host plant chemicals, suggest altered gene expression is important for host race adaptation in *T. conura* . However, expression plasticity is limited, with plastic responses to cross‐fostering limited to the ancestral host race. Further investigation of both different developmental timepoints and exposure lengths will improve our understanding of how expression plasticity is evolving during the ongoing speciation of these fly lineages. Finally, our findings support the role of genomic architecture in the ecotype‐specific gene expression profiles, as consistently DE genes were more densely located within the large, putative inversion on LG3. Whether the overrepresentation of DE genes within the inversion is due to breakpoint‐induced dynamics or results from selection acting within the inversion is an outstanding question, making these incipient species an excellent system for future studies on the architectural and mechanistic basis of adaptive differential expression.

## Author Contributions

A.R. conceptualised and secured funding for the study. J.O.G. designed and carried out experiments, collected samples and extracted RNA for the samples used in the study. O.V.A. provided guidance for RNAseq experimental design and O.M. helped develop RNA extraction methods for *Tephritis conura* larvae. R.A.S. conducted bioinformatic and statistical analyses with contributions from S.C. and Y.S. R.A.S. drafted the manuscript with A.R. All authors contributed to subsequent drafts of the manuscript with comments and suggestions.

## Conflicts of Interest

The authors declare no conflicts of interest.

## Supporting information


Appendix S1

## Data Availability

RNA sequence data have been archived on the European Nucleotide Archive (Steward 2024). Gene predictions and functional annotations, as well as all scripts necessary for analysis and visualisation of the data, are available on GitHub (https://github.com/rstewa03/Tconura_expressionPlasticity.git; Steward 2025).
